# Supplementary material for: Association between oral microbiome and seven types of cancers in East Asian population: a two-sample Mendelian randomization analysis
Source: Front Mol Biosci. 2023 Nov 21;10:1327893. doi: 10.3389/fmolb.2023.1327893 (PMC10702768; doi:10.3389/fmolb.2023.1327893)
Supplement: Supplementary file 1 [file DataSheet1.ZIP › Supplementary Materials/MR plots of saliva/saliva═╝/Prostate cancer/pheno.722_to_prostate cancer_leave_one_out.pdf]

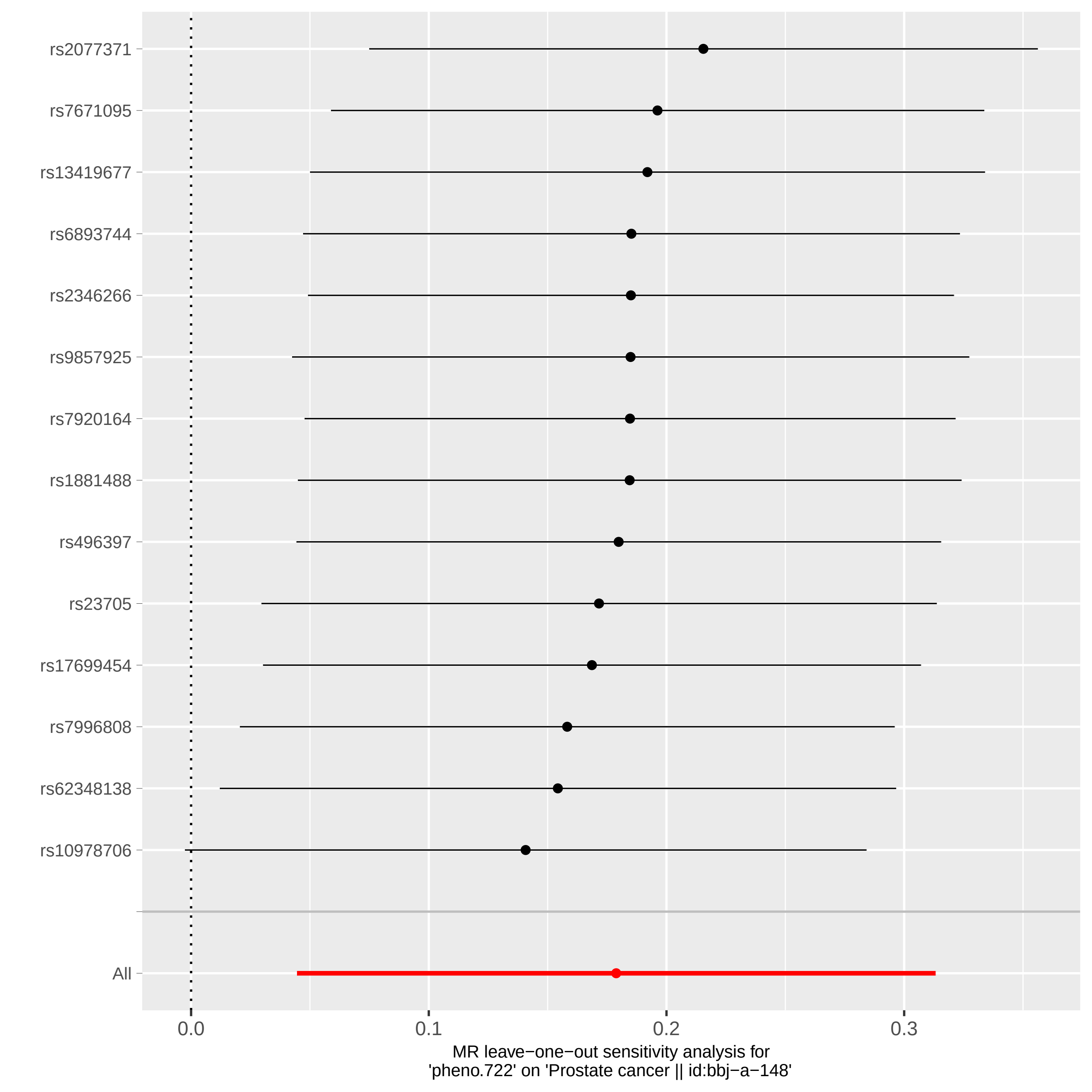
rs2077371
rs7671095
rs13419677
rs6893744
rs2346266
rs9857925
rs7920164
rs1881488
rs496397
rs23705
rs17699454
rs7996808
rs62348138
rs10978706
All
0.0
0.1
0.2
0.3
MR leave−one−out sensitivity analysis for
'pheno.722' on 'Prostate cancer || id:bbj−a−148'
